# Embryos, cancers, and parasites: Potential applications to the study of reproductive biology in view of their similarity as biological phenomena

**DOI:** 10.1002/rmb2.12447

**Published:** 2022-02-11

**Authors:** Yoshihiko Araki

**Affiliations:** ^1^ Institute for Environmental & Gender‐specific Medicine Juntendo University Graduate School of Medicine Chiba Japan; ^2^ Department of Obstetrics & Gynecology Juntendo University Graduate School of Medicine Tokyo Japan; ^3^ Division of Microbiology and Immunology Department of Pathology and Microbiology Nihon University School of Medicine Tokyo Japan

**Keywords:** cancer, embryo, parasite, reproductive biology, viviparity

## Abstract

**Background:**

At present, there are so many living things on the earth. Most of these organisms have a reproductive strategy called sexual reproduction. Among organisms that reproduce sexually, mammals have an extremely complex and seemingly unnatural method of reproduction, or viviparity.

**Methods:**

As an approach to understanding the nature of viviparity, the author have tried to outline the common life phenomena of embryos, cancers, and parasites based on the literature to date, with internal parasites as the keyword.

**Main findings:**

Embryo, cancer, and parasite are constituted as a systemic interaction with the host (mother). Based on these facts, the author proposed the hypothesis that in the case of mammals, "the fetus is essentially harmful to the mother", and that the parasitic fetus grows by skillfully evading the mother's foreign body exclusion mechanism.

**Conclusion:**

Comparative studies of "embryos", "cancers", and "parasites" as foreign bodies have the potential to produce unexpected discoveries in their respective fields. It is important to consider the evolutionary time axis that the basic structure of our mammalian body arose over 200 million years from the Mesozoic Triassic, the period immediately after the Paleozoic Era, when life on Earth became massively extinct.

## Graphical abstract

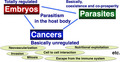

## 1 INTRODUCTION

On a personal note, the author is originally an obstetrician/gynecologist. After graduating from medical school, the author majored immunology in Graduate school, Yamagata University, Japan. At that time, my mentor in the basic science, Professor Fujiro Sendo was a young up‐and‐coming cancer immunologist. He is one of the first researchers to report the natural killer activity of naïve lymphocytes against cancer cells in the 1970s.^1^, ^2^, ^3^ Since there were various regulations in the Japanese national universities in the 1980s, the Department named as "Parasitology", but in reality, it was a mixture of immunologists and parasitologists. Immunology is of course one of the most important aspects of parasitology, but at that time there were still some people doing fieldwork in the Japanese parasitological community. As such, as a beginner the author had many opportunities to attend lectures from researchers in obstetrics and gynecology, cancer immunology, and parasitology, respectively. By the time of my postgraduate studies completed in such an environment, the author found himself thinking, “Embryos, tumors, and parasites are very similar"! more and more often. Interestingly, few researchers, even those with a high degree of expertise in their respective fields, were able to sort out these three areas.

One of the main characteristics of mammals is, as the name suggests, breastfeeding.^4^, ^5^, ^6^ An equally important feature is the reproductive strategy of viviparity (pregnancy), in which a "foreign" fetus is parasitized and nurtured in the mother's body,^7^ although there are rare exceptions such as some sharks and reptiles.^8^, ^9^ The formation of the placenta is necessary for a normal pregnancy to take place and is symbolic of the mammalian reproductive strategy of "internal parasitism".The fertilized egg is the ultimate stem cell, capable of differentiating into any organ, but even embryonic stem cells, the most famous pluripotent stem cells, cannot differentiate into a placenta. Again, in mammals, the only pluripotent stem cells capable of differentiating into all organs are the early embryos before the blastula stage. In nature, therefore, these cells are the basis for the individual life processes of mammals, including the ability to develop into the placenta.

In the light of these scientific discoveries, the author outlines viviparity as a reproductive strategy in mammals, in terms of in vivo cancers and externally introduced parasites. On this basis, the possibility of proposing a new concept to elucidate the nature of reproduction from the findings of each research field is discussed.

## 2 CANCER‐RELATED ANTIGENS AND REPRODUCTION

Classically, so‐called onco‐fetal antigens such as alpha‐fetoprotein and carcinoembryonic antigen are well known, but most of the molecules currently used clinically as tumor markers are glycosylated antigens, and specific changes in glycan structure can be seen early in the carcinogenic process in some cases.^10^, ^11^, ^12^ Since the early 1970s, when lectins were reported to prevent in vitro fertilization,^13^, ^14^ the importance of glycans in sperm‐egg mutual recognition and fertilization has been widely reported in vertebrate including mammals.^15^, ^16^, ^17^, ^18^ However, since glycans are not a direct product of genes and there are many optical isomers, it is difficult to analyze the bioactivity of glycans themselves. Therefore, the bioactivity of glycans is still a matter of controversy in the field of reproductive physiology. This situation is similar to that in cancer biology.

Metastasis is one of the major characteristics of cancer cells. In particular, it is well known that each type of cancer has its own preferred metastatic site.^19^, ^20^, ^21^, ^22^ Whether it is hematogenous or lymphatic metastasis, how do cancer cells that have left the primary site and are wandering around the body recognizing the organ they are in? Simple metastasis formation physically trapped in capillaries or lymph nodes might be considered. However, the existence of favorable metastatic sites, which are unique to each type of cancer, suggests that there are intercellular recognition mechanisms between the cancer cells and their metastatic destination tissues. In cancer, from the perspective of its developmental mechanism, it has recently been revealed that malignant tumors are not simple immortalized tumors, but form a special environment called "microenvironment" between the developing matrix and metastases.^23^, ^24^ This is reminiscent of how the fetus, while foreign to the mother, infiltrates and proliferates into the mother's body after implantation in the endometrium, affecting various cells in the area, while the fetus cross talks with the mother's endocrine and immune systems.^25^, ^26^, ^27^

**FIGURE 1:**
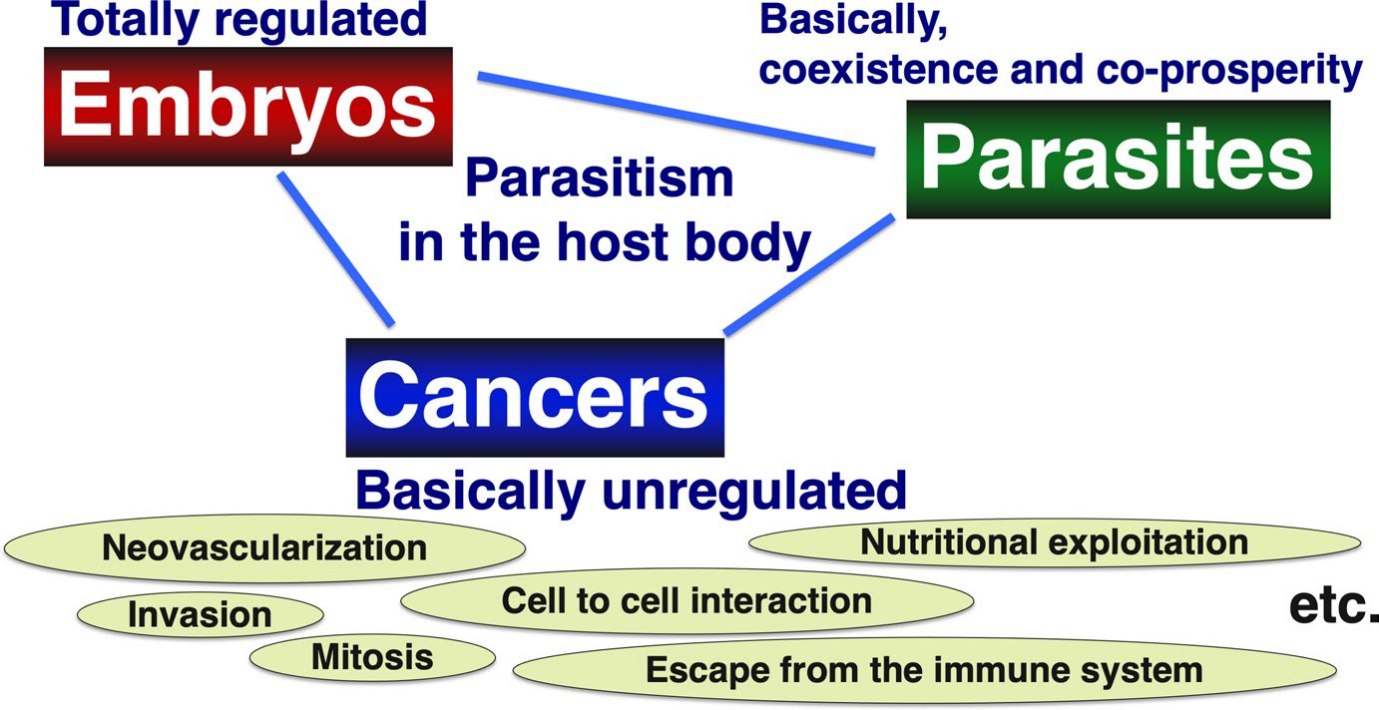
Common biological features found in embryos, cancers, and parasites. Embryos are thought to share a variety of biological phenomena similar to cancer and parasites, and to establish intrapartum parasitism. This parasitic mechanism is presumed to be tightly controlled by the embryo itself and the mother, but its disruption may lead to the nature of various obstetric diseases

On the other hand, many antigen molecules with unknown functions have long been known as cancer‐testis antigens, a group of molecules commonly found in tumor cells and testes.^28^, ^29^, ^30^ It is known that their expression is restricted to the testis, undifferentiated developing cells, and placenta in normal individuals, suggesting that these molecules play an important role in the reproductive process, especially in the gametogenesis. Based on this assumption, many of these groups of molecules classified as cancer‐testis antigens may be involved in their proliferation and survival in cancer cells.^31^ As aforementioned, the fetus (including the placenta) and cancer cells have many things in common in terms of being foreign to the body (Figure 1). In order for the fetus to survive and continue to grow until it is able to adapt to life outside the body, it must have many characteristics in common cell biological characteristics as various cancer cells, including maternal invasion, immune evasion, and angiogenesis. This similarity between germ cells and cancer cells has been pointed out before.^32^, ^33^, ^34^, ^35^, ^36^, ^37^, ^38^ However, these similarities are mainly based on the idea of using the characteristics of cells involved in reproduction (including fetal cells as well as germ cells) for the clinical treatment of cancer. Therefore, there should be a shift in thinking and the idea of using the characteristics of cancer cells for the study of reproduction. This idea has great potential to provide important insights into the elucidation of unsolved mechanisms in reproduction.

## 3 SIMILARITIES IN PARASITE AND REPRODUCTIVE BIOLOGY

There may be a preconceived notion that microbes, viruses, and parasites that cause so‐called infectious diseases are "bad for health". However, this is not necessarily true, and many of them are based on misconceptions. Why are these misconceptions so prevalent?For example, Semmelweis' advocacy of hand disinfection for the prevention of puerperal fever^39^ (translated in English by Carter KC, 1983) resulted in a correct method of prevention, but its methodology was based on empirical rules of observation, which led to persecution based on misunderstanding. The microorganisms themselves had already been discovered at that time.^40^ Later, Pasteur, Koch, and others focused on the study of microorganisms as pathogens, including parasitic diseases, in the late 19th and 20th centuries, and the villainous image of pathogens underlying these infectious diseases seems to have been established (as a side note, Lister's sterilization method^41^ was not persecuted because of the research base of Pasteur and others, and became established in the world). However, it is now well known that many of these viruses, microbes, and parasites are considered to be symbiotic and useful, or merely parasitic. In general, parasites as pathogens satisfy the conditions shown in Table 1 in principle. Based on these principles, if we consider the fetus from a "parasitological" point of view, we can understand the commonality of (1) to (3) rather naturally, but the problematic point of view exists in (4) (Table 1).

**TABLE 1:** Characteristics of parasites as pathogens

Items of general characteristics that the parasite has:
1) It deprives the host of a source of nutritional intake that it should have
2) It is inside or on or near the surface of the host
3) This relationship lasts for a period of time
4) The host suffers a distinct disadvantage from its presence

As already mentioned, pregnancy is a physiological phenomenon characteristic for mammals, as is "breast‐feeding". It is generally considered that there are at least 9 million species of plants, animals, and other living things on the earth,^42^ and most of them reproduce sexually by creating "gametes" of which fusions produce the next generation.^7^ Compared to asexual reproduction, sexual reproduction requires a much higher level of cellular and molecular mechanisms from gametogenesis to fertilization. In addition, more complex and sophisticated mechanisms are required to form the placenta and to maintain the "gestational mechanism" called intrapartum parasitism.

Does the fetus, like a parasite as a pathogen, "bring a definite disadvantage to the mother"? It is usually assumed that the mother nurtures the fetus with compassion. For humans, whose major characteristic is brain activity, "maternal love", symbolized by the Virgin Mary, is a concept that is generally accepted in our society and culture. However, what about the animal nature of the primate, hominid organism? Why, for example, do bears give birth to very small fetuses that are physiologically premature (this is not what Portmann meant by physiological premature birth^43^)? Why do mammals, like marsupials, give birth to small fetuses and raise them in an external parental sac? Furthermore, why did not most non‐mammalian organisms on earth select viviparity when they reproduce sexually? Even parasites are oviparous in their reproduction!

## 4 ON THE UNNATURALNESS OF VIVIPARITY: ITS NATURE FROM THE PERSPECTIVE OF EVOLUTIONARY TIME

As mentioned above, the author believe that we can see the outline of the fact that embryo, cancer, and parasite have quite a few characteristic properties in common in terms of their interrelationship with the host life phenomenon. In other words, cancer, embryo, and parasite present a systemic interaction with the host (mother).

Based on these facts, the author proposes a hypothesis: In the case of mammals, "the fetus is essentially a detriment to the mother", and the mechanisms of embryonic development should share many of the same mechanisms as those used by parasites to coexist with their hosts. The fetus, a parasite, develops while skillfully circumventing the mother's foreign body exclusion mechanism, and leaves the mother when it is ready to survive outside the body. The mother tries in every way possible to control the fetal development during pregnancy so that it does not develop in a disorderly fashion like a cancer. Taking human beings as an example, it is not often that we see a "tumor" that grows from a single cell (fertilized egg) to a weight of more than 4 kg including the fetus and its appendages in just 266 days of repeated differentiation and growth. However, it is thought that the fetus and the mother control the disordered growth of the fetus through the placenta and by using the immune system, endocrine system, and other cellular immunological mechanisms throughout the body.^25^, ^26^, ^27^ If we consider that the end point of this struggle is delivery, and that when the equilibrium is broken in the middle of this struggle, this is when various obstetric diseases such as gestational hypertension and fetal growth retardation occur, the nature of these diseases may become clearer.

**FIGURE 2:**
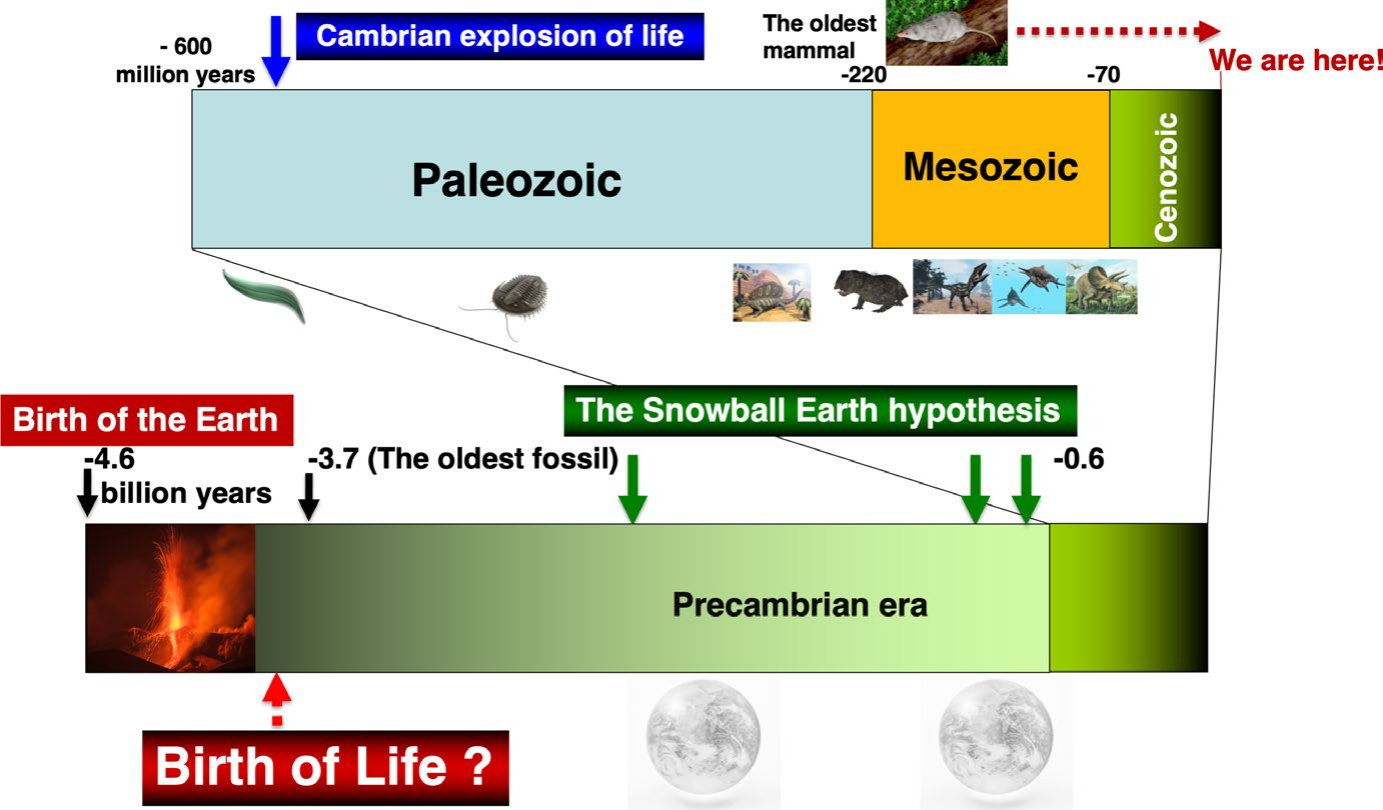
History of the Earth. In a process of life phenomenon formation that spans over 3.5 billion years, the ancestors of mammals are thought to have appeared 200 million years ago. The basic biological characteristics of mammals, such as lactation and placenta formation, have been formed over such a long time. In contrast, human history is only "a short time, close to the moment"

It is widely considered that the ancestors of mammals first appeared on Earth during the Triassic period of the early Mesozoic (when white rot fungi appeared and wood became able to decompose, lowering the partial pressure of oxygen, while the partial pressure of carbon dioxide increased, causing warming)^44^ (Figure 2). Although this period was not always favorable for mammals, mammals achieved mammary gland development as well as embryonic development during this period,^6^, ^45^, ^46^, ^47^ and both of these phenomena have been attributed to the function of independent endogenous retroviruses (ERVs).^48^, ^49^ ERVs are a group of mobile genetic factors called transposons, which contain retrovirus‐like sequences that may fall into the same category as RNA viruses such as SARS‐CoV2, are thought to have infected and assimilated into the cells of organisms during the long process of biological evolution, resulting in the existence of mammals today. In other words, these ERV sequences in the mammalian genome are considered to be traces of past viral infections and invasions. However, not all ERVs are retroviral in origin, as there are examples where such sequences may have left the gene and become infectious on their own, affecting other cells. On the other hand, it is possible that ERV gene sequences are also the source of new retroviruses.^50^

In recent years, analysis of the large number of retrovirus infections and their defense mechanisms in our ancestors has shown that viruses and mammalian cells have co‐evolved in an "arms race" during mammalian evolution to the present day.^51^ If we consider the evolutionary process on this basis, we can vaguely see where the similarities between "fetus, cancer, and parasites" originate from, and we can think of an infinite number of new avenues for future research.

## 5 CONCLUDING REMARKS

One of the traditional Japanese performing arts is a genre of storytelling called "*Rakugo*".^52^ *Rakugo* is the art of storytelling in which a single Rakugo performer plays several characters by oneself. Among these genres, it seems that "sandaibanashi" is the most difficult. This is a type of rakugo in which the audience is asked to come up with three words or titles suitable for a rakugo story, and then the three titles are folded into the story and improvised. In most cases, titles are solicited during the intermission, and the last performer (called "tori"), who is the leader of the performance, has about 2 h to create the piece before his turn. Therefore (as a matter of course), this art is extremely difficult and requires a high level of professional skill, and not many professional *Rakugo* performers are capable of performing it.

"Embryos, Cancers, and Parasites" is an incredibly difficult topic to generate for a "sandaibanashi". Unlike *rakugo*, however, the story is not produced by imagination and composition alone. Therefore, we are now at a crossroads where we can either produce a story with a "punchline" through diligent research or we can waste our time and effort if we misjudge the point of view.

Comparative studies of the Embryos, cancers, and parasites as foreign bodies in the body have the potential to generate unexpected discoveries and concepts in their respective fields. Among other things, it is important to consider the evolutionary timeline that the basic structure of our mammalian body arose over 200 million years from the Triassic of the Mesozoic Era, just after the Paleozoic Era, when most of the organisms on Earth became extinct. For example, with regard to malignant tumors, there have been many reports of malignant tumors in lower vertebrates such as fish, amphibians, and reptiles as well as birds and mammals, while the occurrence of malignant tumors in invertebrates has rarely been reported, even though some species are known to have longevity. While it is well known that there is a strong relationship between cancer development and immune surveillance,^53^ we may now be looking at part of a larger life phenomenon that leads to the hypothesis that mammals took "viviparity" as a reproductive strategy, which may have made an evolutionary trade‐off in increased susceptibility to malignancy.^54^

## CONFLICT OF INTEREST

The author declares no competing financial interests.
